# Development of health‐related quality of life and symptoms of anxiety and depression among persons diagnosed with cancer during adolescence: a 10‐year follow‐up study

**DOI:** 10.1002/pon.3965

**Published:** 2015-09-10

**Authors:** Malin Ander, Helena Grönqvist, Martin Cernvall, Gunn Engvall, Mariann Hedström, Gustaf Ljungman, Johan Lyhagen, Elisabet Mattsson, Louise von Essen

**Affiliations:** ^1^ Clinical Psychology in HealthcareDepartment of Public Health and Caring Sciences, Uppsala UniversityUppsalaSweden; ^2^ Pediatrics, Department of Women's and Children's HealthUppsala UniversityUppsalaSweden; ^3^ Caring Sciences, Department of Public Health and Caring SciencesUppsala UniversityUppsalaSweden; ^4^ Department of StatisticsUppsala UniversityUppsalaSweden

## Abstract

**Objective:**

The main aim was to investigate the development of health‐related quality of life (HRQOL) and symptoms of anxiety and depression in a cohort diagnosed with cancer during adolescence from shortly after up to 10 years after diagnosis.

**Methods:**

Participants (*n* = 61) completed the SF‐36 and the HADS shortly; six, 12, and 18 months; and two, three, four, and 10 years (*n* = 28) after diagnosis. Polynomial change trajectories were used to model development.

**Results:**

Polynomial change trajectories showed an initial increase which abated over time into a decrease which abated over time for the SF‐36 subscales Mental Health and Vitality; an initial decline which abated over time into an increase for HADS anxiety; and an initial decline which abated over time into an increase which abated over time for HADS depression. The SF‐36 mental component summary showed no change from two to 10 years after diagnosis whereas the SF‐36 physical component summary showed an increase from two years after diagnosis which declined over time. Ten years after diagnosis 29% reported possible anxiety.

**Conclusions:**

Development of HRQOL and symptoms of anxiety and depression appears to be non‐linear among persons diagnosed with cancer during adolescence. Well into permanent survivorship an increase in symptoms of anxiety is shown and approximately a third of the participants report possible anxiety. The findings indicate the need for: studies designed to pinpoint the times of highest psychological risk, clinical follow‐up focusing on psychological problems, and development of effective psychological interventions for survivors of adolescent cancer. © 2015 The Authors. Psycho‐Oncology published by John Wiley & Sons Ltd.

## Introduction

Adolescents diagnosed with cancer are not only confronted with the cancer disease and associated stressors but also physical, social, emotional, and wider psychological changes related to the transition from childhood to adulthood 1, 2. Because of frequent hospital stays, aggressive therapies, and impairing side‐effects most adolescents diagnosed with cancer are set apart from peers and everyday life while becoming more dependent on parents than before the disease 3. These circumstances can make it difficult to achieve developmental tasks typically attained during adolescence 2. After end of treatment, survivors face new challenges such as fear of recurrence, returning to school and work, and living with late effects 3, 4. In addition, the onset of many mental health disorders peaks during adolescence and young adulthood 5. In short, facing a cancer diagnosis during adolescence can be extraordinarily challenging 6, potentially having an impact on health‐related quality of life (HRQOL) and causing psychological distress such as symptoms of anxiety and depression.

The majority of long‐term follow up studies on HRQOL and psychological distress among survivors of childhood cancer have demonstrated no or small differences between survivors and healthy peers or population norms [e.g. 7, 8, 9, 10, 11, 12]. However, some studies have shown a lower level of HRQOL and a higher level of psychological distress among persons diagnosed with cancer during adolescence compared to community controls 13, 14 and persons diagnosed at a younger age 15, 16. A recent review concluded that survivors of childhood cancer and controls report comparable psychosocial function, but subgroups, e.g. survivors of central nervous system tumors, are at risk for negative psychosocial late effects 17. Methodological limitations, including small sample sizes, a tendency not to differentiate between children and adolescents 18, use of disputed control groups, and attrition 17, 18 hamper firm conclusions from existing studies. Importantly, a majority of studies are based on cross‐sectional designs 18, 19 and do not allow conclusions regarding development over time with regard to HRQOL and psychological distress. In a study with a longitudinal design Brinkman et al. 20 found that a subgroup of survivors of childhood cancer reported persistent and increasing psychological distress from five years up to decades after diagnosis. To the best of our knowledge, no study has investigated development of HRQOL and/or psychological distress from diagnosis into long‐term survivorship among persons diagnosed with cancer during adolescence. Increased knowledge in the area is necessary to, in regular healthcare, implement appropriate screening procedures at appropriate times to identify potential problems with HRQOL and psychological distress. Moreover, increased understanding of the development of these problems can inform the development of relevant, age‐appropriate psychological interventions matching the needs of the population 20, and thereby reduce human suffering and optimize allocation of resources.

We have reported levels of HRQOL and symptoms of anxiety and depression for a cohort of persons diagnosed with cancer during adolescence from shortly after up to four years after diagnosis in comparison to a matched reference group 21, 22. Findings from these studies show that up to six months after diagnosis the cohort reported lower levels of mental health and vitality and a higher level of symptoms of depression than the reference group 21, 22. Four years after diagnosis the cohort reported a higher level of vitality and a lower level of symptoms of anxiety and depression than the reference group 22. The main aim of this study is to investigate development of HRQOL and symptoms of anxiety and depression in the cohort from shortly after up to 10 years after diagnosis. A secondary aim is to identify the proportion reporting possible anxiety and/or depression from shortly after up to 10 years after diagnosis. We chose to focus on possible anxiety and depression as subclinical symptoms of anxiety and depression are associated with significant suffering which can be alleviated if identified and addressed within regular healthcare.

## Methods

### Design

Participants completed questionnaires assessing HRQOL (SF‐36) and symptoms of anxiety and depression (the Hospital Anxiety and Depression Scale) shortly (four to eight weeks) (T1); six (T2), 12 (T3), and 18 (T4) months; and two (T5), three (T6), four (T7), and 10 (T8) years after diagnosis. Data collected at T1–T7 have been reported 21, 22. In this study data from T1 to T8 are presented to illustrate development of HRQOL and symptoms of anxiety and depression from shortly after up to 10 years after diagnosis.

### Sample

Swedish speaking adolescents 13–19 years diagnosed with cancer for the first time or with a recurrence after having been off treatment for at least one year; treated with chemotherapy; and cognitively, emotionally, and physically able to participate were eligible and included 1999 to 2003 at three of the six Swedish pediatric oncology centers. A nurse assessed eligibility in collaboration with a physician. During the inclusion period, 90 adolescents were diagnosed with cancer for the first time and 10 with a recurrence, 11 were not eligible (five did not speak Swedish, five were considered physically unable to participate, and one was lost because of administrative reasons). Of the remaining 65 who agreed to participate, two became too ill before T1 and two were lost because of administrative reasons. Fifty‐six newly diagnosed adolescents and five with a recurrence were included, rendering a participation rate of 69%, 19 of these participated at all assessments. Clinical and demographic characteristics at T1–T8 and reasons for attrition at T2–T8 are presented in Table 1. At T8, 19 were deceased, seven had withdrawn, and seven could not be reached. At T8 all participants (*n* = 28) were off treatment.

**Table 1:** Clinical and demographic characteristics for the cancer group at T1–T8 and reasons for attrition at T2–T8

	T1 *n* = 61	T2 *n* = 56	T3 *n* = 50	T4 *n* = 48	T5 *n* = 38	T6 *n* = 42	T7 *n* = 39	T8 *n* = 28
Diagnosis								
CNS tumor	3	3	3	3	1	2	2	2
Ewing sarcoma	4	3	2	2	1	1	1	0
Leukemia	21	17	14	13	12	12	10	8
Lymphoma	20	20	20	20	15	17	18	12
Osteosarcoma	8	8	6	6	5	6	5	4
Other	5	5	5	4	4	4	3	2
Treatment status								
On/Off	61/0	42/14	14/36	11/37	9/29	4/38	1/38	0/28
Age, years								
Mean	15.5	16.0	16.7	17.2	18.0	18.7	19.8	25.3
(SD)	(1.7)	(1.6)	(1.6)	(1.6)	(1.6)	(1.7)	(1.7)	(1.6)
Gender								
Male	37	35	29	28	22	23	21	15
Female	24	21	21	20	16	19	18	13
Attrition								
Death	—	1	7	2	2	0	3	4
Withdrawal from all further participation	—	1	1	0	3	0	0	2
Refrained from participation at one assessment	—	3	1	1	0	0	1	0
Administrative reasons^a^	—	0	0	0	6	2	1	7

a Administrative reasons included: could not be reached and not contacted at T5 because of delayed ethical approval.

T1 = four to eight weeks after diagnosis (DI); T2 = six months after DI; T3 = 12 months after DI; T4 = 18 months after DI; T5 = two years after DI; T6 = three years after DI; T7 = four years after DI; T8 = 10 years after DI.

### Measures

HRQOL was measured with the Short Form 36 (version 1.0) (SF‐36) measuring: Physical Functioning (PF); Role Physical (RP); Bodily Pain (BP); General Health (GH); Vitality (VT); Social Functioning (SF); Role Emotional (RE); and Mental Health (MH) 23. One summary scale measures physical health: the Physical Component Summary (PCS) and one summary scale measures mental health: the Mental Component Summary (MCS) 24. PCS is a measure of PF, RP, BP, and GH, whereas MCS encompasses VT, SF, RE, and MH. SF‐36 is adequate from early adolescence 23, 25 and valid and reliable for use with survivors of childhood cancer 26. Response choices vary from two to six, raw scores are transformed from 0 (worst possible) to 100 (best possible) 27. PCS and MCS scores are standardized to a mean of 50, a score above 50 representing better than average and below 50 poorer than average 27.

Symptoms of anxiety and depression were measured with the Hospital Anxiety and Depression Scale (HADS) 28 which includes two subscales, one measuring anxiety and one depression, each consisting of seven items. Items are rated from 0 (no distress) to 3 (maximum). Subscale scores range from 0 (no distress) to 21 (maximum). There are two ways to interpret scores; either via a comparison to normative values/values for a reference group or using a cut‐off. A cut‐off of eight on each subscale to identify possible (sub‐clinically relevant) and 11 to identify probable (clinically relevant) anxiety and depression has been recommended for adults 28. HADS use for adolescents 12–17 years has been validated in a non‐clinical sample 29; a cut‐off of seven to identify possible depression and nine to identify possible anxiety was recommended. To minimize false negatives and positives we used a cut‐off of seven to identify possible depression and nine to identify possible anxiety among participants 13–17 years and a cut‐off of eight to identify possible anxiety and depression among older participants.

### Procedure

The study was approved by the regional Ethics Review Board at the Faculties of Medicine at the universities of Lund, Umeå, and Uppsala.

Approximately three weeks after diagnosis, eligible adolescents and their parents were provided oral and written information by a nurse. Informed consent was asked for and for those 13–17 years parental consent was requested. Participants answered questions in the SF‐36 subscales MH and VT at T1–T4, all SF‐36 subscales at T5–T8, and the HADS subscales at T1–T8. It took five to 10 min to answer the questions. Most background data was collected at T1. All data was collected via telephone between 1999 and 2013.

### Statistical analysis

Mixed models were used to investigate initial status and development over time for the SF‐36 and HADS subscales. Intercepts and slopes were included as random effects. Polynomial change trajectories were used to investigate development over time by linear, quadratic, and cubic terms, and models were chosen based on model fit. Unconditional models were tested and gender, age, and leukemia vs. not‐leukemia were subsequently added as potential predictors. Simulation studies indicate that multi‐level modeling, of which mixed models with longitudinal data is an example, produces unbiased regression coefficients even with small sample sizes such as the current 30. Independent two‐tailed t‐tests were used to investigate possible differences between responders and non‐responders at T8 for the SF‐36 and HADS subscales at T1 and T7. α < 0.05 was considered as indicating a significant difference. All analyses were performed in IBM SPSS Statistics 20^©^.

## Results

**Figure 1:**
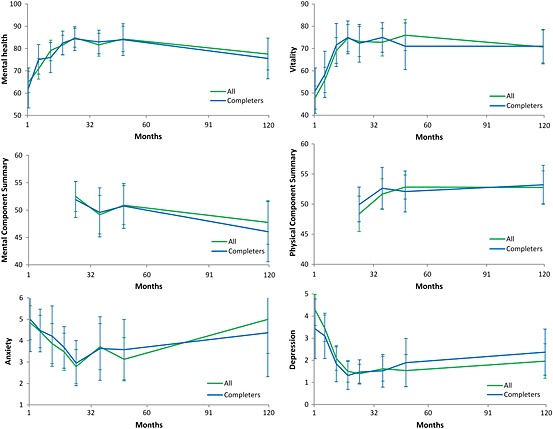
Observed means and 95% confidence intervals for the study variables shortly after up to 10 years after diagnosis

Clinical and demographic characteristics at T1–T8 and reasons for attrition at T2–T8 are presented in Table 1 whereas descriptive statistics for study variables are presented in Table 2. Development over time for study variables is presented in Figure 1.

**Table 2:** Descriptive statistics for study variables

	T1 *n* = 61 M (SD)	T2 *n* = 56 M (SD)	T3 *n* = 50 M (SD)	T4 *n* = 48 M (SD)	T5 *n* = 38 M (SD)	T6 *n* = 42 M (SD)	T7 *n* = 39 M (SD)	T8 *n* = 28 M (SD)
SF‐36 Mental Health	65.0 (18.8)	70.9 (17.2)	79.1 (16.2)	81.6 (15.3)	84.8 (12.9)	81.7 (16.5)	84.3 (17.7)	77.6 (18.4)
SF‐36 Vitality	48.0 (20.7)	55.9 (21.9)	69.1 (20.8)	74.7 (21.7)	73.2 (21.3)	72.9 (20.5)	76.0 (22.2)	70.7 (20.6)
SF‐36 Mental Component Summary	—	—	—	—	52.5 (8.6)	49.2 (11.7)	50.9 (11.3)	47.7 (10.8)
SF‐36 Physical Component Summary	—	—	—	—	48.4 (9.3)	51.7 (8.4)	52.8 (6.3)	52.8 (6.3)
HADS Anxiety	4.8 (3.2)	4.4 (2.9)	3.9 (3.4)	3.5 (3.1)	2.8 (2.5)	3.7 (3.6)	3.1 (3.2)	5.0 (4.3)
HADS Depression	4.3 (2.8)	3.4 (2.3)	2.1 (1.9)	1.5 (1.7)	1.4 (1.3)	1.6 (1.8)	1.5 (2.3)	2.0 (2.1)

SF‐36, Short Form 36; HADS, Hospital Anxiety and Depression Scale. T1 = four to eight weeks after diagnosis (DI); T2 = six months after DI; T3 = 12 months after DI; T4 = 18 months after DI; T5 = two years after DI; T6 = three years after DI; T7 = four years after DI; T8 = 10 years after DI.

Final models of initial status and change over time are presented in Table 3. For MH and VT a cubic model provided best fit to the data, showing an initial increase which abated over time into a decrease which abated over time. For MCS an unconditional means model with no change over time provided best fit to the data. A visual inspection (see Table 2 and Figure 1) suggests a cubic development; however at least five time‐points are needed to estimate a cubic growth model 31. For PCS a quadratic model provided best fit to the data suggesting an increase from T5; the rate of increase declined over time. For HADS anxiety, a quadratic model provided best fit to the data showing an initial decline; the decline abated over time into an increase. There was a trend for girls to initially report a higher level of anxiety compared to boys. For HADS depression a cubic model provided best fit to the data showing an initial decline which abated over time into an increase which abated over time. Girls had a more pronounced initial decline, subsequent increase, and final decrease.

**Table 3:** Final models of initial status and change over time for study variables

	Shape	Initial status (intercept)	Linear term (slope)	Quadratic term (slope)	Cubic term (slope)	Predictors of initial status	Predictors of change
SF‐36 Mental Health	Cubic	64.87***	13.29***	−2.73***	0.14***	—	—
SF‐36 Vitality	Cubic	47.80***	19.90***	−4.20***	0.22***	—	—
SF‐36 Mental Component Summary	No change	50.24***	—	—	—	—	—
SF‐36 Physical Component Summary	Quadratic	48.90***	1.81**	−0.15**	—	—	—
HADS Anxiety	Quadratic	4.13***	−0.54***	0.05***	−	Girl (Est. = 1.39, *p* = .06)	—
HADS Depression	Cubic	4.22***	−1.64***	0.27**	0.10*	—	Girl × Linear term (Est. = −1.07*)
							Girl × Quad term (Est. = 0.41**)
							Girl × Cubic term (Est. = −0.30**)

SF‐36, Short Form 36; HADS, Hospital Anxiety and Depression Scale.

* *p* < 0.05

** *p* < 0.01

*** *p* < 0.001

At T1 18% and at T8 none reported possible depression; at T1 15% and at T8 29% reported possible anxiety. Four of eight persons reporting possible anxiety at T8 reported possible anxiety at one or more previous assessments. Of the remaining 20 persons nine reported possible anxiety at one or more previous assessments. There were no significant differences between responders at T8 and non‐responders at T8 for any of the SF‐36 and HADS subscales at T1 and T7 (t‐values ranging from 0.00 to 1.76 and p‐values from 1.00 to 0.08).

## Discussion

To the best of our knowledge this is the very first study to report development of HRQOL and symptoms of anxiety and depression in a cohort diagnosed with cancer during adolescence from shortly after diagnosis up to permanent survival. Development of HRQOL as well as symptoms of anxiety and depression appears to be non‐linear. The first phase of survivorship was characterized by a low level of HRQOL; thereafter an increasing level of HRQOL and a decreasing level of symptoms of anxiety and depression were shown up to four years after diagnosis. The findings suggest a decreasing level of HRQOL and an increasing level of symptoms of anxiety from four to 10 years after diagnosis. Ten years after diagnosis almost a third reported possible anxiety. This result supports previous findings showing that a sub‐group of survivors of childhood cancer report clinically relevant psychological distress decades following diagnosis 13, 20 as well as a conclusion from a recent meta‐analysis 32 showing that symptoms of anxiety rather than depression are a problem for long‐term survivors of adult cancer.

When interviewed 10 years after diagnosis the cancer group reported negative cancer‐related consequences, among these health worries; frustration about healthcare; and fertility concerns 33 not reported previously during the disease trajectory. This suggests that permanent survivorship of adolescent cancer is associated with specific challenges. A recent study showed that cancer survivors 25–29 years of age reported a higher level of symptoms of anxiety and depression and a lower level of physical and emotional wellbeing than those 30–39 years 34. The results were explained by younger survivors facing more interpersonal, vocational, and financial challenges while having a less stable social network than older survivors 34. It is reasonable to assume that the family acts as an important buffer against psychological distress during the first years after an adolescent has been diagnosed with cancer. When the survivor leaves home and enters permanent survival the family's buffering effect may diminish while new stable relationships have not always been established. This circumstance can increase vulnerability to developmental and cancer‐related stressors. Others have reported a delayed social development for survivors of childhood cancer, differences in family and living conditions compared to controls, and that survivors are less likely to marry or be in a partnership than the general population 13, 35, 36.

The findings of non‐linear development and the high individual variability in possible anxiety case‐ness between assessments indicates that it is difficult to predict who will suffer reduced HRQOL and/or psychological distress during the extended survival phase. This indicates a need of long‐term monitoring of the psychological health of individuals treated for cancer during adolescence to detect those in need of psychological support supporting recent guidelines for the psychosocial care of individuals treated for childhood cancer 37, 38. However, future research is needed to examine whether such monitoring is feasible as well as acceptable for the target population.

This study has methodological strengths including a high recruitment rate, a longitudinal design covering 10 years, and a high retention rate considering the time span. In spite of this, the fact that only 28 persons participated 10 years after diagnosis may hamper conclusions with regard to development over time of HRQOL and symptoms of anxiety and depression. However, the fact that there were no differences between responders and non‐responders for any variable shortly after and/or four years after diagnosis supports the validity of the findings. Yet, the risk of biased estimates needs to be considered and the risk of type II errors must be kept in mind. Furthermore, survivors of a central nervous system (CNS) tumor were underrepresented which may have had an impact on findings as a CNS tumor diagnosis is associated with poor HRQOL and psychosocial problems 17, 35, 39. Another fact to consider is that data was collected via telephone. It has been shown that older adolescents and adults report a higher level of HRQOL and a lower level of psychological distress via telephone than via postal questionnaires 40, 41, 42. When considering development over time with regard to investigated variables the issue is not a problem as all data was collected via telephone. However, when considering the proportion reporting possible anxiety and/or depression it should be noted that the proportions might have been higher had data been collected via postal questionnaires. Using the HADS to screen for anxiety and depression has been questioned because of inconsistencies regarding its factor structure 43, 44. The majority of studies support a two‐factor solution 45, 46 and the HADS has performed well in assessing symptom severity and identifying anxiety and/or depression disorders in somatic populations 47. However, the optimal cut‐off to identify possible anxiety and/or depression has not been identified for the present population and the risk of over‐ or underestimation of possible case‐ness should be kept in mind when interpreting the results. More importantly, the HADS might only capture a part of the cancer‐related psychological suffering that survivors of adolescent cancer may experience. How to best conceptualize this suffering remains to be determined. Last, existing Swedish norm data for the HADS 48 differ from the data presented in this paper with respect to age of subjects, when data were collected, and method of administration, all of which preclude meaningful direct comparison. The lack of appropriate comparison data impedes interpretation of the findings as it cannot be concluded whether the observed development of symptoms of anxiety and depression is related to cancer survivorship or simply reflects normal development from adolescence to young adulthood. Norm‐data collected via mail in 1991–1992 for SF‐36 at different ages for the general Swedish population suggest for women and men respectively a decrease in physical HRQOL with increasing age, whereas mental HRQOL appears stable across the life span 27. No differences between age groups 15–19, 20–24, and 25–29, i.e. the relevant age groups for this study, were reported for any subscale 27. The facts that reported norm data was collected via mail whereas data reported herein was reported via telephone and that data presented in this report was collected up to approximately 23 years after norm data preclude direct comparisons between the two data sets; however available norm data indicates that the observed development of HRQOL for persons diagnosed with cancer during adolescence is not an effect of normal development.

## Conclusion

Development of HRQOL and symptoms of anxiety and depression appears to be non‐linear among persons diagnosed with cancer during adolescence. Well into permanent survivorship an increase in symptoms of anxiety is shown, and approximately a third of the participants report possible anxiety. The findings indicate the need for: studies designed to pinpoint the times of highest psychological risk, clinical follow‐up focusing on psychological problems, and development of effective psychological interventions for survivors of adolescent cancer.

## Conflict of interest

The authors declare no conflicts of interest.
